# Improved Xenobiotic Metabolism and Reduced Susceptibility to Cancer in Gluten-Sensitive Macaques upon Introduction of a Gluten-Free Diet

**DOI:** 10.1371/journal.pone.0018648

**Published:** 2011-04-12

**Authors:** Karol Sestak, Lauren Conroy, Pyone P. Aye, Smriti Mehra, Gaby G. Doxiadis, Deepak Kaushal

**Affiliations:** 1 Division of Microbiology, Tulane National Primate Research Center, Covington, Louisiana, United States of America; 2 Department of Microbiology and Immunology, Tulane University School of Medicine, New Orleans, Louisiana, United States of America; 3 Division of Comparative Pathology, Tulane National Primate Research Center, Covington, Louisiana, United States of America; 4 DNA Microarray and Expression Core, Division of Bacteriology and Parasitology, Tulane National Primate Research Center, Covington, Louisiana, United States of America; 5 Comparative Genetics and Refinement, Biomedical Primate Research Centre, Rijswijk, The Netherlands; Florida International University, United States of America

## Abstract

**Background:**

A non-human primate (NHP) model of gluten sensitivity was employed to study the gene perturbations associated with dietary gluten changes in small intestinal tissues from gluten-sensitive rhesus macaques (*Macaca mulatta*).

**Methodology:**

Stages of remission and relapse were accomplished in gluten-sensitive animals by administration of gluten-free (GFD) and gluten-containing (GD) diets, as described previously. Pin-head-sized biopsies, obtained non-invasively by pediatric endoscope from duodenum while on GFD or GD, were used for preparation of total RNA and gene profiling, using the commercial Rhesus Macaque Microarray (Agilent Technologies),targeting expression of over 20,000 genes.

**Principal Findings:**

When compared with normal healthy control, gluten-sensitive macaques showed differential gene expressions induced by GD. While observed gene perturbations were classified into one of 12 overlapping categories - cancer, metabolism, digestive tract function, immune response, cell growth, signal transduction, autoimmunity, detoxification of xenobiotics, apoptosis, actin-collagen deposition, neuronal and unknown function - this study focused on cancer-related gene networks such as cytochrome P450 family (detoxification function) and actin-collagen-matrix metalloproteinases (MMP) genes.

**Conclusions/Significance:**

A loss of detoxification function paralleled with necessity to metabolize carcinogens was revealed in gluten-sensitive animals while on GD. An increase in cancer-promoting factors and a simultaneous decrease in cancer-preventing factors associated with altered expression of actin-collagen-MMP gene network were noted. In addition, gluten-sensitive macaques showed reduced number of differentially expressed genes including the cancer-associated ones upon withdrawal of dietary gluten. Taken together, these findings indicate potentially expanded utility of gluten-sensitive rhesus macaques in cancer research.

## Introduction

Gluten sensitivity is one of the prominent features of Celiac disease (CD) which is an autoimmune disorder characterized by damaged lining of the small intestine due to responses triggered by dietary gluten, a protein complex found in wheat, rye, and barley [2]. A series of histopathological manifestations were described in celiac patients and also in rhesus macaques with gluten-sensitive enteropathy – a newly established non-human primate model of gluten sensitivity [1], [3], [4], [5]. In humans, Marsh [6], [7] divided CD-associated lesions, based on severity, into five categories. In rhesus macaques, lesions were described to range between mild intraepithelial and lamina propria lymphocytosis up to almost complete flattening and fusion of the small intestinal villi, with chronic inflammation of the corresponding lamina propria [1], [5]. Gluten-sensitive macaque model was recently used to demonstrate the functional changes in intestinal permeability during the stage of epithelial disrepair caused by dietary gluten. It was found that at the stage of disrepair, a gluten-derived peptide such as alpha-2-gliadin 33-mer is capable of penetrating beneath intestinal epithelium into lamina propria, consistent with findings described in celiacs [3], [4], [5], [8]. Disrupted pattern of intestinal tight junctions and deposition of autoantibodies targeting the enzyme that plays a vital role in gluten digestion i.e. tissue tranglutaminase 2 (TG2) in duodenum of macaques with gluten enteropathy but not in healthy controls was also noted [5].

Despite extensive study of clinical, immunological and histopathological properties of CD, relatively little is known about the effects of gluten sensitivity on other diseases and their potential relationship. Lack of knowledge is apparent when trying to study the factors that trigger the transition of one stage of the disease into another. Underlying genetic predisposition that only began to be elucidated in gluten-sensitive macaques is another important component of this complex disease. Due to a growing number of clinical reports that suggest association between CD and intestinal T cell lymphomas, adenocarcinomas, diabetes type 1, Autism or Down syndrome [9], [10], [11], [12], [13], [14], [15], [16], it is important to establish if observations from above studies can also be demonstrated at the molecular level. Such research should provide novel leads for existing therapies of not only CD but also aforementioned diseases. In order to study the selected gene networks in gluten-sensitive primate host, a quantitative microarray gene technology and rhesus macaque model were used. It was hypothesized that perturbations in cancer-associated gene networks would be seen in gluten-sensitive animals at the stage of relapse versus stage of remission. Minimal to no perturbations induced by dietary gluten changes were expected in normal healthy control and in gluten-sensitive animals at the stage of remission.The primary goal was to show that gluten-sensitive primates respond to dietary gluten changes by up/down-regulation of selected genes and that such perturbations can be linked to an increased risk to other diseases such as cancer. Secondary goal was to determine if withdrawal of dietary gluten is sufficient to reverse these changes.

## Results

### Increased levels of plasma AGA and TG2 antibodies in gluten-sensitive macaques

**Figure 1:**
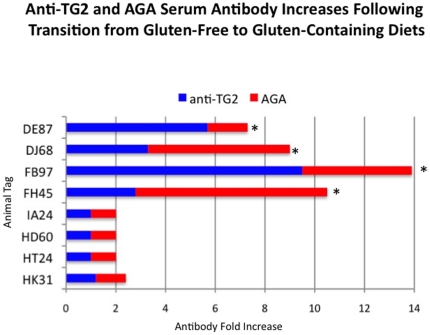
AGA and anti-TG2 antibodies in plasma. Plasma antibody responses to gliadin and TG2 were measured in four gluten-sensitive (FH45, FB97, DJ68 and DE87) and four normal healthy control (HK31, HT24, HD60 and IA24) rhesus macaques at the time of remission and/or relapse. Graph depicts fold-increases of antibodies over the baseline upon introduction of dietary gluten. Significant (p<0.05) increases are indicated (*).

Withdrawal of dietary gluten resolves in gluten-sensitive macaques clinical, histopathological as well as immunological manifestations of the disease including the decline of AGA and TG2 antibodies in plasma [1], [3]. In human patients, combined presence of AGA and TG2 antibodies is considered to be a highly accurate predictor of CD [2]. In this study, transition from GFD to GD was accompanied by significant (p<0.05) increase of both AGA and TG2 antibodies in gluten-sensitive macaques (Figure 1). In accord with these and previous reports, the GFD period with baseline levels of AGA and TG2 antibodies is indicated here as the stage of immunological remission while the GD period with significant increase of AGA and TG2 antibodies, is indicated as the stage of immunological relapse. In normal healthy control macaques, AGA and TG2 antibodies remained at baseline levels regardless of the dietary gluten intake.

### Differential gene expression in duodenum of gluten-sensitive macaques

**Figure 2:**
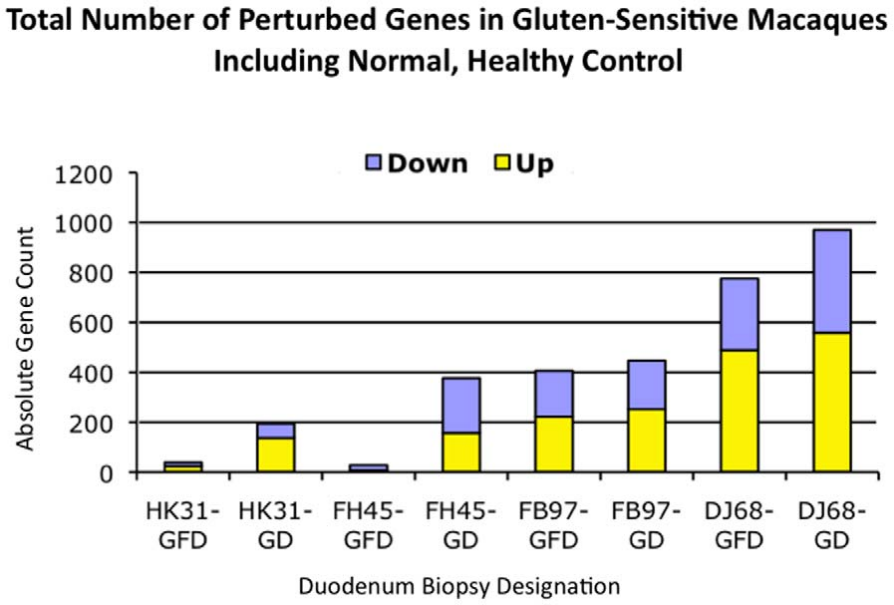
Differential gene expression. A commercial rhesus-specific gene array was used to measure the differential gene expression in eight duodenum samples from four selected study macaques: Normal healthy control (HK31), andFH45, FB97 and DJ68 gluten sensitive macaques at the stages of remission and/or relapse. Absolute numbers of perturbed genes out of ∼20,000 tested per each sample are shown.

In comparison with normal healthy control, gluten-sensitive macaques showed an increased number of differentially expressed genes during the stage of relapse while on GD (Figure 2). Although all three tested gluten-sensitive animals showed a lower number of differentially expressed genes during the stage of remission, FH45 macaque was the only one that normalized its gene expression during the GFD treatment to a baseline level – comparable with that of a healthy control HK31 (Figure 2). This was not entirely unexpected, since time needed to accomplish the remission might substantially differ in between different individuals, as it is influenced by many physiological and genetic factors [17], [18].

### Categories of differentially expressed genes

**Figure 3:**
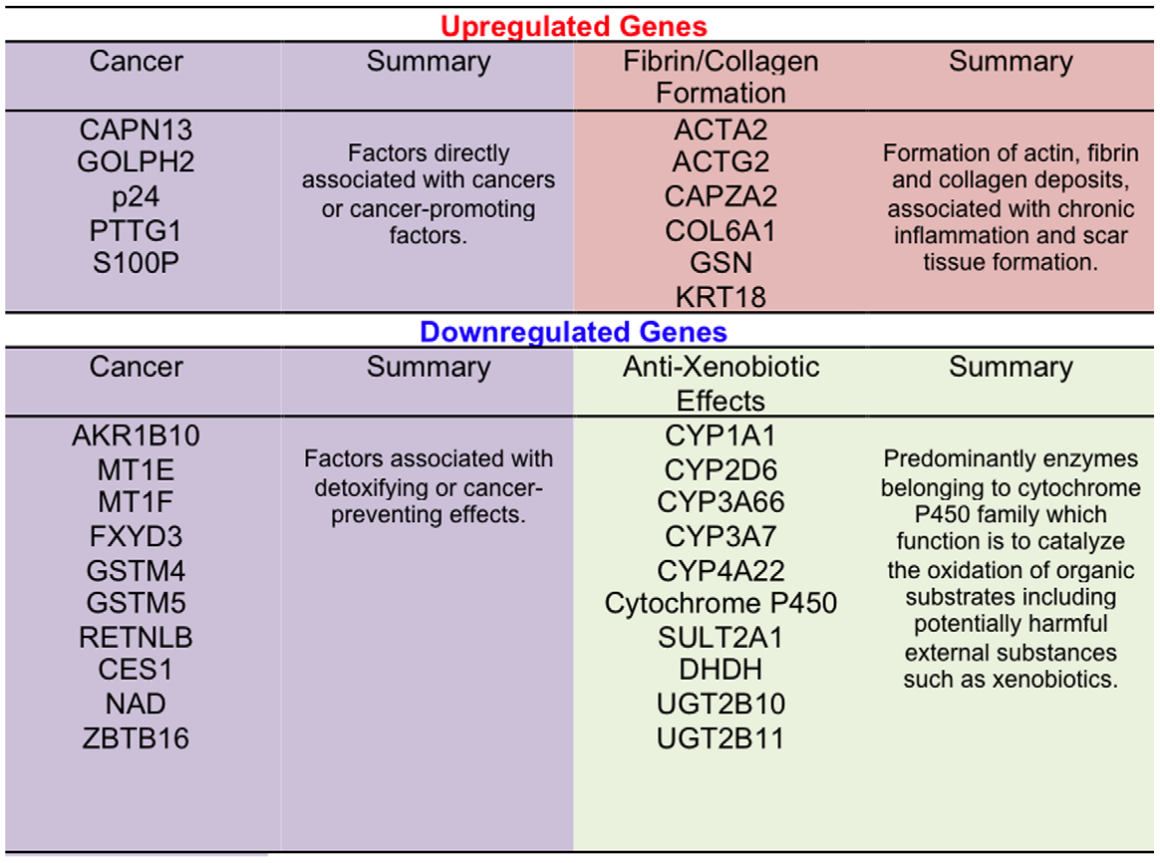
FH45 macaque. Selected three categories of perturbed genes in duodenum of FH45 gluten-sensitive macaque (normalized against normal, healthy control macaque HK31) while on gluten diet. Different color shades correspond with four categories of perturbed genes. Similar patterns of gene perturbation were identified also in FB97 and DJ68 gluten-sensitive macaques (not shown).

**Figure 4:**
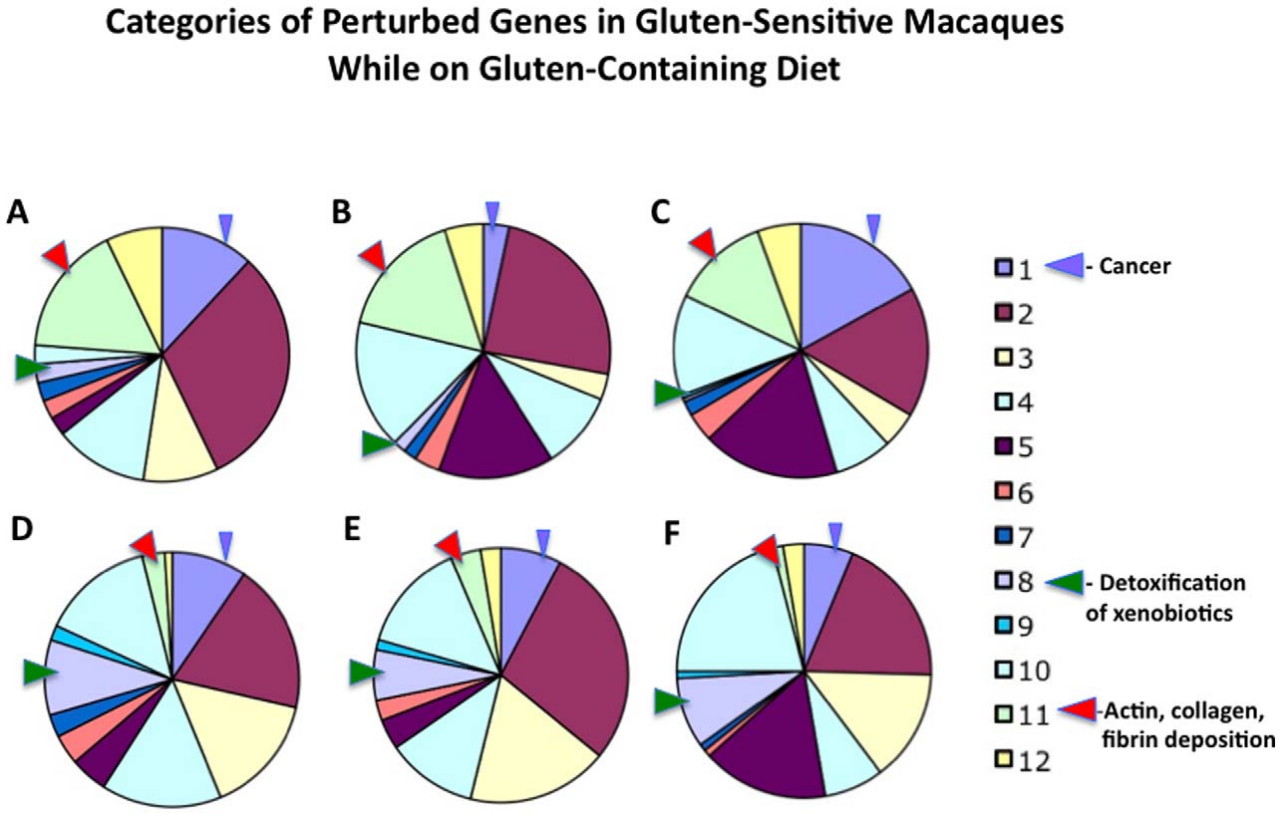
Categories of differentially expressed genes in gluten-sensitive macaques during the relapse stage. 1 – cancer, 2 – metabolism, 3 – digestive tract function, 4 – immune response, 5 – cell growth, 6 – signal transduction, 7 – autoimmunity, allergy, 8 – detoxification of xenobiotics, 9 – apoptosis, 10 – not known function, 11 – actin-collagen-MMP network, and 12 – neuronal function. Relative distributions of up-regulated gene categories are shown in panels A (FH45), B (FB97) and C (DJ68) while down-regulated genes are shown in panels D (FH45), E (FB97) and F (DJ68).

**Figure 5:**
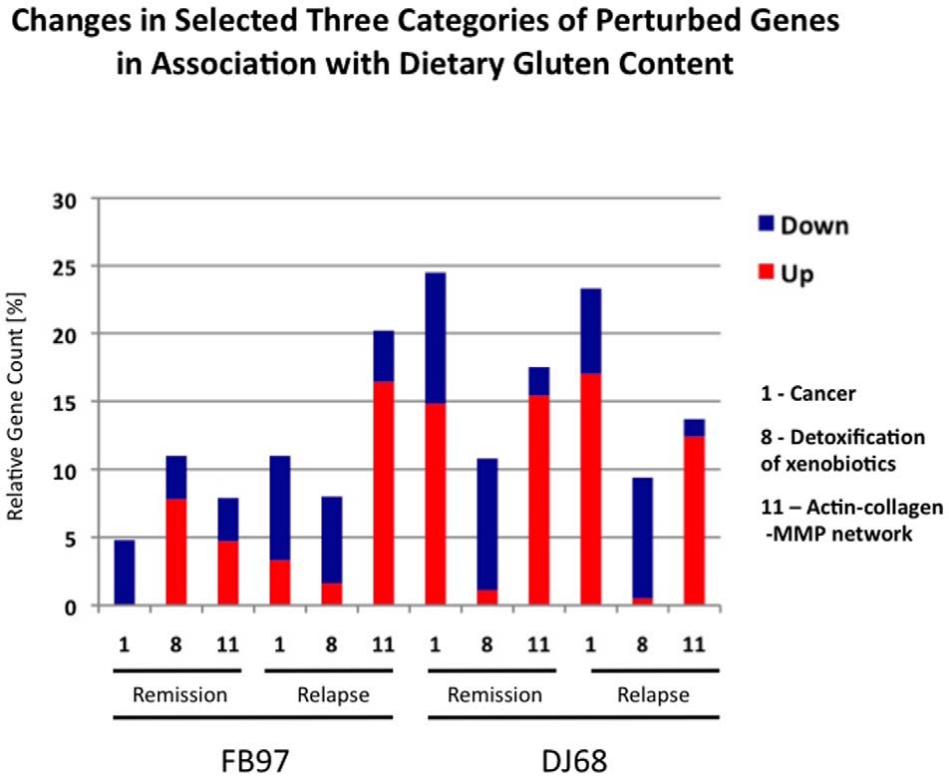
Dietary gluten withdrawal and differential gene expression. Dietary gluten withdrawal impacts relative distribution of perturbed gene categories in gluten-sensitive macaques. In three selected categories [1 (cancer), 8 (detoxification of xenobiotics), and 11 (actin-collagen-MMP network)], these changes included decreased number of “cancer-promoting” events such as down-regulation of category 8 genes and up-regulation of category 11 genes. Data generated with FB97 and DJ68 macaques during the stage of remission and relapse are shown. FH45 macaque had only few gene perturbations during the stage of remission while on gluten-free diet, thus relative gene counts are not shown for this animal.

All genes with differential level of expression were further divided into 12 overlapping categories: 1- cancer, 2- metabolism, 3- digestive tract function, 4- immune response, 5- cell growth, 6- signal transduction, 7- autoimmunity, allergy, 8- detoxification of xenobiotics, 9- apoptosis, 10- not known function, 11- actin-collagen-MMP gene network, and 12- neuronal function. Three categories subject to closer examination included: cancer, detoxification of xenobiotics and actin-collagen-MMP gene network. These categories were selected based on their direct or indirect involvement in cancer progression or suppression (Figures 3 and 4). Since cancer (category 1) genes were represented by two distinct subcategories i.e. cancer-promoting and cancer-suppressing and both of these subcategories contained up- and down-regulated genes, category 1 ended up with less distinct pattern of differential expression than category 8 or 11 (Figures 4 and 5). Categories 8 and 11 showed predictable pattern of differential expression in all three gluten-sensitive macaques: While genes responsible for detoxification of xenobiotics were predominantly down-regulated during relapse (Figures 3 and 4D,E, F), genes of actin-collagen-MMP network were up-regulated (Figures 3 and 4A,B, C).

### Impact of dietary gluten withdrawal on differential gene expression

Although there were similarities in gene expression of three gluten-sensitive macaques during the relapse stage (Figure 4), there were also differences during the remission stage(Figure 5). The most likely explanation for the differences is that despite that all three gluten-sensitive animals were in immunological remission at the time of biopsy procedure, the FB97 and DJ68 macaques' differential gene expressions were not yet at baseline, in spite of spending 10 weeks on GFD. This was more apparent in case of DJ68 than in FB97, while complete baseline and no perturbations were seen in FH45 macaque during the remission stage while on GFD (Figure 2). Predictably, introduction of dietary gluten resulted in increase of perturbed genes in three tested gluten-sensitive animals with DJ68 being the highest (Figures 2 and 5).

### Cytochrome P450 family gene network is down-regulated in gluten-sensitive macaques upon introduction of dietary gluten

**Figure 6:**
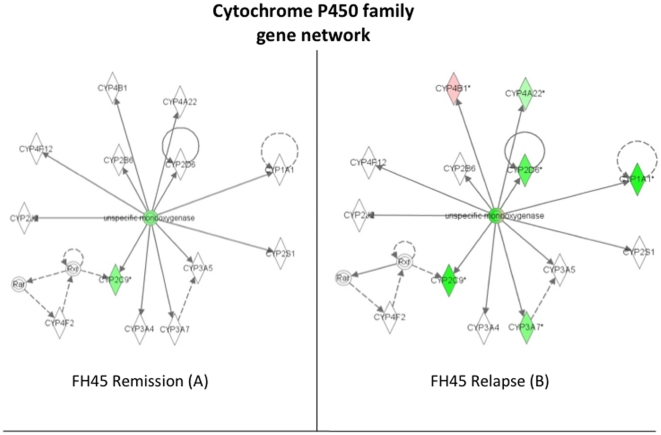
Down-regulation of cytochrome P450 family gene network. Introduction of dietary gluten (relapse stage) down-regulates several of the genes in P450 network while dietary gluten withdrawal (remission stage) normalizes its expression towards baseline level: Expression in FH45 gluten-sensitive macaque shows 5 out of 6 perturbed genes being more down-regulated during the relapse stage than during the remission stage. Red signifies up-regulation of a gene while green signifies down-regulation, with the standard level of expression determined by HK31 normal healthy control macaque. Intensity of color represents the magnitude of change in expression. The solid lines mean that the gene's protein product directly affects the target gene's protein levels, whereas dotted lines mean that the gene's protein product indirectly alters the target gene's protein levels through the involvement of an intermediate gene.

Based on observations with celiac patients who show significant down-regulation of CYP3A4 gene, a member of the P450 family, as well as an increased incidence of adenocarcinoma and lymphoma of the small intestine, it was predicted that gluten-sensitive macaques would show down-regulation of P450 family genes while on GD. They were expected to have relatively normal expression of these genes while on GFD. Differential expression of P450 gene network in all three gluten-sensitive macaques followed above prediction. The P450 gene network of FH45 animal is shown for illustration (Figure 6): Six out of seven perturbed genes in the P450 network were down-regulated at the stage of relapse in contrast to only two at the stage of remission. The fact that one (CYP4B1) gene was up-regulated in FH45 during the stage of relapse illustrates the complex relationship among factors associated with gluten sensitivity. Since CYP4B1 metabolizes carcinogens, its function is especially important in the presence of multiple cancer-promoting factors.

### Actin-collagen-MMP gene network is differentially expressed in gluten-sensitive macaques upon introduction of dietary gluten

**Figure 7:**
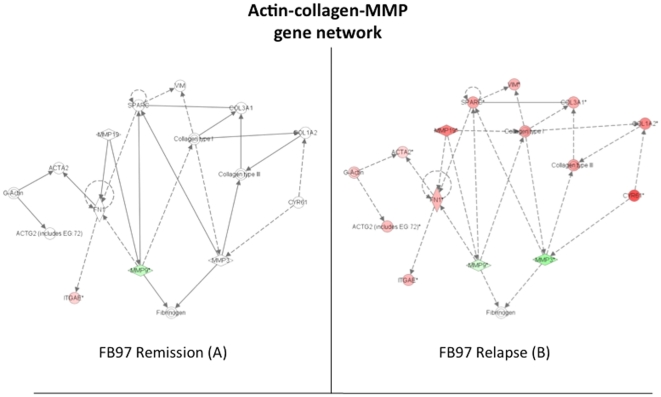
Perturbations in the actin-collagen-MMP gene network. Introduction of dietary gluten (relapse stage) up-regulates most of the actin and collagen genes while MMP3 and MMP9 are down-regulated. Data generated with FB97 gluten-sensitive macaque during the stage of remission and relapse are shown. Red signifies up-regulation of a gene while green signifies down-regulation, with the standard level of expression determined by HK31 normal healthy control macaque. Intensity of color represents the magnitude of change in expression. The solid lines mean that the gene's protein product directly affects the target gene's protein levels, where as dotted lines mean that the gene's protein product indirectly alters the target gene's protein levels through the involvement of an intermediate gene.

This network regulates the expression of actin and collagen that are important for the wound healing process and the scar tissue formation. An up-regulation of these genes was expected as a possible compensatory mechanism to repair the tissue damage caused by chronic inflammatory process and/or auto-immune reaction in the small intestine. Although FH45 and DJ68 gluten-sensitive animals showed several of these genes being up-regulated at the stage of relapse (not shown), the difference with the stage of remission was not as prominent as it was in case of FB97 where almost the entire network was perturbed during the stage of relapse (Figure 7). While actin and collagen differential gene expression was consistent with expected up-regulation and/or scar tissue formation, the two key matrix metalloproteinases MMP3 and MMP9 were down-regulated, contradicting the above “scar tissue formation” hypothesis.

### Several disease-associated gene groups are differentially expressed in gluten-sensitive macaques

**Figure 8:**
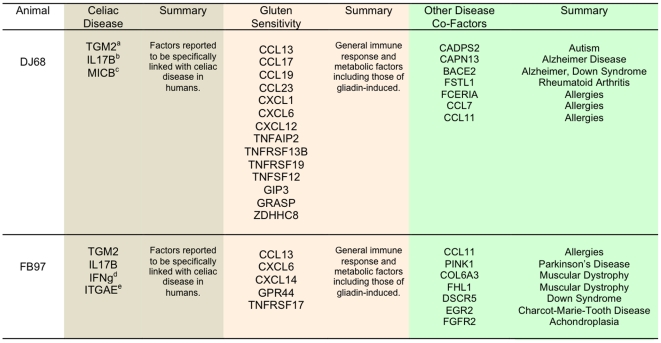
DJ68 and FB97 macaques. Selected categories (a. Celiac Disease, b. general gluten sensitivity or inflammation and c. other disease) of upregulated genes in duodenum of DJ68 and FB97 gluten-sensitive macaques (normalized against normal, healthy control macaque HK31) while on gluten-containing diet. Different color shades correspond with different categories of perturbed genes. ^a^TGM2 = tissue transglutaminase 2 functions as celiac disease autoantigen; ^b^IL17B = IL-17 cytokine reported to be produced in celiacs but not in other gluten-sensitive patients; ^c^MICB = NKG2D ligand suggested to mediate the transformation of TCR-dependent CD8+ CTLs to TCR-independent (NK-like) effector IELs in celiac patients; ^d^IFNg = interferon gamma is produced by T cells and promotes gluten peptide flux across intestinal epithelium; ^e^ITGAE i.e. CD103 antigen was suggested to mediate the celiac-specific autoimmune response in the small intestine.

In addition to 12 categories of perturbed genes identified in this study in gluten-sensitive macaques (Figure 4), it was of interest to identify genes that would link this condition with other diseases than cancer, as increasingly reported in clinical studies involving celiacs [9], [10], [11], [12], [13], [14], [15], [16]. All three tested gluten-sensitive macaques showed differential expression of several genes that could be linked with not only CD but also with Autism, food allergy, Down Syndrome, Alzheimer Disease, Rheumatoid Arthritis, Parkinson's Disease and Muscular Dystrophy (Figure 8). Although significance of these findings needs to be investigated further, it provides potentially novel direction for future research regarding the development of non-human primate models of above diseases.

### *Mamu II* composition of gluten-sensitive macaques

Although all four gluten-sensitive macaques assigned for this study had antibodies during the relapse stage against not only gliadin but also TG2 (autoantigen), their *Mamu II* (*DQA1*, *DQB1* and *DRB*) allele composition proved to be not uniform (Table 1). This possibly indicates that gluten sensitivity is in rhesus macaques either a) associated with more than one *MHC II* allele due to a higher diversity and a broader repertoire of *Mamu II* than *HLA II* [19], [20] or b) gluten-sensitivity is in macaques not genetically-linked with *Mamu II*. Additional studies are currently in progress with larger number of gluten-sensitive macaques to elucidate this issue.

**Table 1:** Composition of *Mamu-DQA1*, *-DQB1* and *-DRB* alleles of four gluten-sensitive macaques used in this study.

Macaque	Mamu-DQA1*	Mamu-DQB1*	*Mamu-DRB*
FH45	*01:05:01/26:01*	*06:02:01/18:01*	*W6:06,*W21:04,*W26:03/1*03:03:01,1*10:07
DJ68	*23:02/26:01*	*18:04/18:01*	*W1:01,*W6:02,*W6:09:01/1*03:03:01,1*10:07
DE87	*01:05:01/05:04*	*06:02:01/17:03*	*W6:06,*W21:04*,W26:03/*W20:02:01,*W25:01
FB97	*01:02/23:02*	*06:05/18:04*	Not defined

Note: The Mamu-DQA1*, -DQB1* and *-DRB* alleles were targeted due to their possible involvement in autoimmunity.

## Discussion

A gluten-sensitive rhesus macaque model was used to study the differential gene expressions induced by GD. In addition, the potential relationship between gluten sensitivity and cancer was examined. Although there is growing number of clinical reports suggesting such relationship in celiac patients, studies that focus on molecular mechanisms linking these conditions are absent. As described in earlier reports, administration of GFD or GD to gluten-sensitive rhesus macaques is associated with absence or presence of series of the clinical, histopathological, metabolic and immunological symptoms, collectively referred to as stages of remission or relapse, respectively [1], [3], [4], [5]. Consistent with our recent study, gluten-sensitive and control macaques were selected from the larger cohort of 500 candidate animals based on presence of plasma AGA and TG2 antibodies [5]. Since combined presence of AGA and TG2 antibodies is considered in human patients as highly reliable predictor of CD [2], [21], it is important to stress that all four gluten-sensitive macaques in this study had both AGA and TG2 antibodies. In contrast to our previous work [1], [5], histopathological signs were not used as the predictors of gluten sensitivity since mucosal damage was not the subject of this study.

Since there is a growing evidence suggesting association between CD, intestinal cancers and other diseases, we took advantage of the rhesus macaque model of gluten sensitivity to elucidate A) if such relationship can be detected on the gene expression level, and B) if changes in dietary gluten intake influence selected gene expression. Towards this end, we mainly utilized a rhesus-specific microarray capable of measuring the expression of over 20,000 genes in one tissue sample. Differentially perturbed genes were classified into 12 overlapping categories including three main categories of interest: cancer, detoxification function, and actin-collagen-MMP genes. In addition, differentially expressed genes that were previously reported to be associated with various other diseases were also identified (Figure 8).

The intestinal cytochrome P450 genes such as CYP3A4 participate in metabolism of xenobiotics to reduce their adverse effects. However, the expression of CYP3A4 in celiac patients is reduced significantly compared to healthy adults [22], [23]. Moreover, CD patients show increased incidence of intestinal T-cell lymphoma and adenocarcinoma [17], [18]. Therefore, the reduced levels of cytochromes P450 might increase the bioavailability of xenobiotics in celiacs, thereby increasing the incidence of xenobiotic-induced cancer progression. In order to determine if above hypothesis can be contemplated in studies with gluten-sensitive rhesus macaques, expression of cytochrome P450 genes along with other “cancer-associated” genes was measured in duodenum biopsies obtained at the stages of remission and relapse. It was predicted that lack of cytochromes P450 gene expression is reversed by withdrawal of dietary gluten – by placing the gluten-sensitive macaques on GFD. Results of this study clearly show that down-regulation of cytochrome P450 gene network occurs in gluten-sensitive macaques while on GD. It was also observed that such down-regulation is ameliorated by GFD treatment although complete reversal to a level comparable with normal healthy individual was accomplished only in one out of three gluten-sensitive macaques tested by microarrays. It is plausible from generated results that if the gluten-sensitive macaques would be kept on GFD for a longer period, remaining two animals would continue reversing cytochrome P450 down-regulation. The cytochrome P450 gene network down-regulation was paralleled with overall up-regulation of actin-collagen-MMP gene network. Consistent with our findings, it was reported that up-regulation of actin and collagen with simultaneous down-regulation of MMP3 and MMP9 occurs as a measure to prevent the cancer formation in experimentally-treated mice [24]. Since this was observed in FB97 macaque during relapse, it is likely that gene perturbations in actin-collagen-MMP network are indicative of a protective innate response against cancer invasion in at least some of the gluten-sensitive primates (Figures 3 and 7).

Despite the predictable patterns of differential gene expression in aforementioned category 8 (detoxification) and 11 (actin-collagen-MMP) genes, category 1 (cancer) did not follow such pattern, due to high complexity of gene interactions in this category, with multiple cancer-promoting and –suppressing genes being simultaneously affected by dietary gluten changes. Taken together with category 8 and 11 gene expression results however, our results indicate that relapse stage of gluten sensitivity in rhesus macaques is conducive to tumorigenesis and it can be partially reversed by withdrawal of dietary gluten.

Many questions still remain. Some of these are due to our incomplete knowledge whether rhesus macaques class II alleles are associated with gluten sensitivity in a fashion analogous to human celiac patients i.e. *DQ2/8* alleles [2], [18], [25], [26]. Our current model can only be referred as “gluten sensitivity” model, keeping in mind that there are at least two categories of gluten sensitivity in primates: first of which is *Mamu II*-dependent (CD-like) while second is *Mamu II*-independent (food allergy-like). Our results involving the genetic analysis of 50+ rhesus macaques with and without gluten sensitivity indicate that a *Mamu II*-associated predisposition for this condition exists in rhesus monkeys[Sestak et al., unpublished]. Relatively high variation in differential gene distribution in this study might thus be explained by different *Mamu II* backgrounds in selected gluten-sensitive animals (Table 1). In order to improve the rhesus model of gluten sensitivity and to make it more relevant as a CD model, selection of gluten-sensitive macaques will need to be carried out in future also in the context of *MamuII* pre-screening. Our results indicate that CD, food allergy and other disease condition-associated genes including the cancer-related ones are differentially expressed in gluten-sensitive macaques. Finally, results reported here show that above gene perturbations are ameliorated and even abrogated by dietary gluten withdrawal. These results can be used as a platform for future studies aiming at elucidation of relationship between CD and cancer.

## Materials and Methods

### Ethics statement

Approval for all veterinary procedures in this study had been obtained from the Tulane University Animal Care and Use Committee (Protocol # 3508), Animal Welfare Assurance A-4499-01. Animals in this project were under the full care of veterinarians of the Tulane National Primate Research Center in accordance with the standards incorporated in the Guide to the Care and Use of Laboratory Animals (NIH) 78-23 (Revised, 1996). All veterinary procedures were performed only with sedated animals. Animal welfare and steps were taken to ameliorate suffering in accordance with the recommendations of the Weatherall report.

### Animals and diets

Four gluten-sensitive (FH45, DJ68, DE87 and FB97) and four normal, healthy control (HK31, HT24, HD60 and IA24) rhesus macaques (*Macaca mulatta*) of Indian subspecies, 2–9 years of age, simian retrovirus-free, of either sex were used in this study. Since prevalence of gluten sensitivity in macaques is similar to that of humans, gluten-sensitive animals were selected from the larger cohort of 500 candidates, as described in detail elsewhere [5]. Gluten-sensitive macaques were distinguished from normal, healthy ones by presence of both anti-gliadin (AGA) and anti-tissue transglutaminase 2 (TG2) serum antibodies. All gluten-sensitive animals were kept at least 10 weeks on gluten-free diet (GFD), followed by 10 weeks of regular, gluten-containing chow (GD) (PMI Nutrition Intl., St. Louis, MO), to accomplish the stage of remission and relapse. The transition of GFD to GD was characterized by significant (p<0.05) increase of AGA and anti-TG2 serum antibodies above the baseline (Figure 1). In case of normal healthy control animals, antibody levels were always in baseline regardless of dietary gluten intake.

### Small intestine and peripheral blood sample collection

Two ml of peripheral blood was collected at the stage of remission and/or relapse from all animals as described [5]. In addition, sample of distal duodenum (five pin-head sized biopsies) was collected at the stage of remission and relapse from three gluten-sensitive animals with highest level of antibody increase (Figure 1) by pediatric endoscope as described [5]. In case of healthy control animal (HK31), endoscopy procedure was also performed twice in parallel with procedures done in gluten-sensitive animals despite that no relapse stage characterized by significant antibody increase was present in HK31 in association with introduction of dietary gluten (Figure 1). All collected biopsy samples were immediately placed in five ml of RNA-later solution (Qiagen, Valencia, CA) to preserve the RNA. Blood was processed to extract the plasma, which was stored at −20°C and tested by indirect ELISA for the presence of anti-gliadin and anti-TG2 antibodies [1].

### RNA isolation, cDNA synthesis

Total RNA was extracted from the intestinal biopsies using the Qiagen'sRNeasy plus micro kit, following the instructions of manufacturer. One pin-head-size biopsy out of five collected was sufficient to isolate the required amount of RNA using the RNeasy kit.NanoDrop spectrophotometer (Thermo Scientific, Wilmington, DE) was used to measure the RNA concentration and purity. Total RNA was used to perform the cDNA synthesis and cyanine dye labeling as described previously [27], using the Agilent Low RNA Input Linear Amplification-Labeling Kit (LRILAK). The labeled cDNA was used to perform overnight hybridization to Agilent's rhesus macaque whole genome microarray, according to the manufacturer's instructions [28].

### Microarray procedure and gene expression

A commercially available oligonucleotide rhesus macaque whole genome Microarray encompassing ∼20,000 target genes (Agilent Technologies, Palo Alto, CA) was used. Arrays were printed using the 4×44 k format (Agilent Technologies). The slides were washed according to the manufacturer's instructions and then imaged using the GenePix Pro 6.1 software. Individual measurements were recorded in at least two duplicates. The resulting .*gal* files were analyzed by SpotfireDecisionSite, Microarray Analysis module (TIBCO-Spotfire, Boston MA) as previously described [28]. Following the removal of unreliable spots, the data was normalized using a Locally Weighted Scatterplot Smoothing (LOWESS) algorithm and the resulting ratios of change in gene-expression captured on a logarithm to two scale [28]. Each set of differentially expressed genes contained both “up-regulated” and “down-regulated” ones. In compliance with MIAME guidelines, the raw microarray data associated with this manuscript will be uploaded to Gene Expression Omnibus database prior to publication.

### Gene function

In order to define and to categorize the function of individual differentially expressed genes, a web-based program www.genecards.org (Weizmann Institute of Science, Rehovot, Israel) was used.

### Analysis of rhesus major histocompatibility class II (*Mamu II*) composition

*Mamu-DRB* typing was performed by using a *DRB* specific microsatellite (D6S2878) as described recently [29]. *DQA1* and *DQB1* alleles have been determined by direct sequencing. Therefore, polymerase chain reactions (PCR) were performed in a mixture of 10 ng DNA (*DQA1*) or 30 ng DNA (*DQB1*), 1×PCR buffer II (Invitrogen), 5 mMdNTPs, 10 uM of each primer (5′ DQA1-intr1b-M13: TGT AAA ACG ACG GCC AGT CCT GCT TGT CAT CTT CAC TCA and 3′ DQA1-GH27: CAC GGA TCC GGT AGC AGC GGT AGA GTT G for DQA1, 5′ DQB1-M13: TGT AAA CGA CGG CCA GTT CCC CGC AGA GGA TTT CGT G and 3′ DQB1-intr 2: TGC GGG CGA CGACGA CGC CTC ACC TC for DQB1, respectively), 50 mM MgCl_2_, and 5 units of *Taq* polymerase (Invitrogen). The cycling parameters for the PCR and the sequencing reactions were the same as described earlier [30]. For allele definition the program SBT engine (Genome Diagnostics, Utrecht, The Netherlands) was used. The nomenclature for the Major Histocompatibility Complex (*MHC*) genes for non-human primates was described by Ellis and coworkers [31] and the allele names were given in accord with recently revised nomenclature for the human MHC [32].

### Statistical analysis

In order to determine if anti-gliadin and anti-TG2 antibody levels increased significantly after introduction of dietary gluten in gluten-sensitive animals above the baseline represented by negative control animals, a Wilcoxon Signed Rank Test with a significance of p<0.05, was used. The microarray experiments were designed so as to normalize the gene expression levels in duodenum samples from gluten-sensitive macaques relative to those obtained from a normal, healthy, control macaque while on gluten-free diet. At least two-fold differences in relative expression, with a significance of p<0.05, were considered differentially expressed (t-test function in SpotfireDecisionSite, Functional Genomics module).
